# Occurrence of Delirium during ECMO Therapy in a Critical Care Unit in Poland—A Cross-Sectional Pilot Study

**DOI:** 10.3390/ijerph18084029

**Published:** 2021-04-12

**Authors:** Sabina Krupa, Adriano Friganovic, Wioletta Mędrzycka-Dąbrowska

**Affiliations:** 1 Institute of Health Sciences, College of Medical Sciences of the University of Rzeszow, Poland St. Warzywna1A, 35-310 Rzeszow, Poland; sabinakrupa@o2.pl; 2 Department of Anesthesiology and Intensive Medicine, University Hospital Centre Zagreb, University of Applied Health Sciences, Mlinarska cesta 38, 10000 Zagreb, Croatia; adriano@hdmsarist.hr; 3 Department of Anaesthesiology Nursing & Intensive Care, Faculty of Health Sciences, Medical University in Gdansk, 80-211 Gdańsk, Poland

**Keywords:** delirium, extracorporeal membrane oxygenation, critical care

## Abstract

Background: The problem of delirium during extracorporeal membrane oxygenation (ECMO) therapy, which has rarely been studied, is an important issue since it is necessary to ensure patient safety during therapy. This study aimed to show the incidence of delirium in patients after extracorporeal membrane oxygenation therapy and factors affecting the occurrence of delirium in this group of patients. Design: A cross-sectional study was conducted. Method: The study involved a group of patients from an intensive cardiac care unit who received extracorporeal membrane oxygenation therapy. The study lasted for more than two years, in the period from 2018 until 2020. The Nursing Delirium Screening Scale (NuDESC) and the Delirium Observation Screening Scale (DOSS) were applied. Additionally, the patients were examined using Numeric Rating Scale (NRS), the Insomnia Severity Index (ISI), the Richmond Agitation Sedation Scale (RASS), the Ramsay Sedation Scale (RSS), and a thirst intensity scale; ultimately, relationships between these factors and delirium were examined. Results: In patients who underwent ExtraCorporeal Membrane Oxygenation (ECMO) therapy, delirium was confirmed by the NuDESC in 68.75% of patients in the evening hours, while it was measured by the DOSS scale in 84.38% of patients in the morning. The study found that ECMO delirium was not associated with hyperactivity, sleep disturbance, sedation, pain, or thirst. Conclusion: Delirium in patients undergoing ECMO therapy was confirmed by both the NuDESC and DOSS. Factors such as thirst and sleep disturbance after ECMO therapy were shown to influence the occurrence of delirium. The diagnosis of delirium using standardized scales is possible provided that more tests are carried out. Research should be conducted to determine whether the NuDESC is equivalent to the DOSS.

## 1. Introduction

Extracorporeal membrane oxygenation (ECMO) therapy is an increasingly common supportive therapy when it comes to the treatment of respiratory and circulatory failure. Sedation is necessary in most patients for safety reasons. When making sedation decisions it is important to find out whether the patient is susceptible to delirium during ECMO therapy. A better understanding of the factors affecting development of delirium reduces the risk of decannulation in stimulated patients [1]. Effects of delirium in intensive care unit (ICU) patients may be severe. The incidence of delirium contributes to increased mortality rates in the affected patients, longer durations of mechanical ventilation, and consequently an extended duration of patient stay in the ICU, finally leading to cognitive impairments in such patients [2]. The American Psychiatric Association’s fifth edition of the Diagnostic and Statistical Manual of Mental Disorders (DSM-5) diagnostic criteria for delirium are as follows: disturbance of consciousness (reduced clarity of awareness of the environment) accompanied by a reduced ability to focus, sustain, or shift attention. The DSM-5 revised the diagnostic criteria for delirium [3]. Hyperactive delirium is characterized by (motor) agitation, restlessness, and sometimes aggressiveness. Hypoactive delirium is characterized by motor retardation, apathy, and slowing of speech, and patients can appear to be sedated. Mixed delirium is a combination of hyperactive and hypoactive delirium [4]. Delirium with no motor symptoms indicates that patients only experience the cognitive symptoms of delirium. The hypoactive subtype seems to be more common than the hyperactive subtype; however, it is less likely to be identified or reported as these patients exhibit fewer behavioral problems and are often perceived as cooperative [4]. The assessment of delirium should be regarded as a standard practice [5]. The main risk factors for delirium identified by researchers include the use benzodiazepines as well as immobilization [6,7]. ICU delirium is underestimated if it is not adequately monitored, and therefore it is underreported due to multiple overlapping difficulties, but it should be screened actively [8]. Delirium monitoring and assessment should be standard practice in ICUs [9]. The scales recommended by the Society of Critical Care Medicine (SCCM) for use in ICUs include the Confusion Assessment Method in the Intensive Care Unit (CAM-ICU) and the Intensive Care Delirium Screening Checklist (ICDSC). In accordance with the recommendations, an official translation of the scales should be applied. The scale officially translated into Polish is the Confusion Assessment Method in the Intensive Care Unit (CAM-ICU) [10]. Unfortunately, no validation study related to the Polish version of CAM and CAM-ICU is currently available. In the responding ICUs, delirium is monitored in only 11.9% of the units in Poland. A sedation monitoring tool is used in only 46.1% of the units. Only 19.4% of ICUs have written protocols for sedation and 32.1% do not practice daily sedation interruption [11].

A literature review showed no similar research on Poland, and as such here a scientific contribution in this field is made. This study aimed to show the incidence of delirium in patients after extracorporeal membrane oxygenation therapy and factors affecting the occurrence of delirium in this group of patients.

## 2. Materials and Methods

### 2.1. Design

A cross-sectional study was conducted. The project was implemented in the Intensive Care Cardiac Surgery Unit.

### 2.2. Setting

This article was written in accordance with the Strengthening the Reporting of Observational Studies in Epidemiology (STROBE) guidelines to improve the quality of reporting of this observational study (File S1) [12]. The study lasted for more than two years in the period from 2018 until 2020. Patients in the Intensive Care Cardiac Surgery Unit who underwent extracorporeal membrane oxygenation qualified for the study. The data collection procedure was started with the consent of the heads of the institutions where the data was collected. The next step, in line with the guidelines of the Helsinki Declaration, was the approval of the Bioethics Committee. After receiving the consent of the Bioethics Committee, a consent form for participation in the study was established, in which the patient/family was informed about the possibility of withdrawing from participation in the study at any stage. In the case of ECMO patients, first consent was given by the family due to the patient’s condition. After awakening, the patient made a decision as to whether he/she still consented to participate in the study. Both families and patients were explained what the research would involve and that it was anonymous and would not affect their health in any way.

Scales for delirium assessment were completed by nurses who were trained and knew how to use delirium assessment tools.

### 2.3. Participants

Detecting delirium is not a standard used in our country. Not every patient meets the criteria for the delirium evaluation protocol. Patients were randomly selected for the study (all ECMO-treated patients who entered ICU). Patients in the Intensive Care Cardiac Surgery Unit who underwent extracorporeal membrane oxygenation qualified for the study. The patients were examined 6 days after the start of ECMO therapy. All patients with venovenous and arteriovenous ECMO were included in the study. Random selection of the patients meant that they were enrolled automatically without prior consultation. All patients undergoing ECMO therapy were examined. The patients were included in the study based on calculations according to the Nursing Delirium Screening Scale (NuDESC), which assesses delirium [13].

### 2.4. Data Sources/Measurement

#### 2.4.1. Scales for Delirium Testing

The Nursing Delirium Screening Scale (NuDESC) is a 5-point observational scale that nurses can use to assess delirium in patients. The NuDESC is a delirium screening instrument that can be easily integrated into routine care and clinical practice. It is easy to use, time-efficient, and accurate, and can lead to prompt delirium recognition and treatment. The NuDESC shows promise as a useful concomitant delirium research tool, allowing continuous screening, symptom monitoring, and severity ratings. The NuDESC is psychometrically valid and has a sensitivity and specificity of 85.7% and 86.8%, respectively [14]. At the next stage, the severity of delirium symptoms was analyzed.

The Delirium Observation Screening Scale (DOSS) was developed to facilitate early recognition of delirium by nurses during routine clinical care. The original version of the scale consisted of 25 items measuring the early symptoms of delirium. Each element of the scale is scored as 0 or 1. If 3 or more points are obtained, delirium in the patient can be discussed. The test takes about 5 min to connect [15]. The DOSS scale has also been successfully used in the ICU [16]. The DOSS was also used in another study on postoperative care. Delirious patients showed higher incidence of adverse events [17].

#### 2.4.2. Scales Assessing the Effect on the Occurrence of Delirium

The Numeric Rating Scale (NRS) is one of the most commonly used pain scales in medicine. It consists of 10 points, where 0 is no pain and 10 is unbearable pain [18].

The Richmond Agitation Sedation Scale (RASS) is an instrument designed to assess the level of alertness and agitated behavior in critically ill patients. It is a scale of 10 degrees ranging from −5 to +4. The points indicate the level of the patient’s arousal, starting with “awakens to voice” and ending with “unarousable.” The scale was created in cooperation with the teams working in the hospital: doctors, nurses, and pharmacists [19].

The Ramsay Sedation Scale (RSS) is a scale used in anesthesiology to assess the depth of sedation in intensive care units (1: agitated; 2,3: comfortable; 4,5,6: sedated) [20].

The Insomnia Severity Index (ISI) is a scale which is composed of 7 items that evaluate the severity of difficulties related to sleep onset, sleep maintenance, and early morning awakening, as well as satisfaction with current sleep pattern, interference with daily functioning, and noticeability of impairment attributed to the sleep problem, in addition to the level of distress caused by the sleep problem.

A result of 0–10 points indicates no insomnia, 11–14 points suggests subliminal insomnia, and a result above 14 points refers to the occurrence of clinically significant insomnia [21]. During the assessment the patients were awake and coherent.

Thirst was rated on a scale from 0 to 10, where 0 is no thirst and 10 is unbearable thirst [22].

Measurements with the use of selected scales were performed on the sixth day after cardiac surgery, in accordance with the requirements of the scale.

In accordance with the recommendations for using individual scales in nursing practice, delirium was assessed twice during the duty cycle using the NuDESC, while the DOSS was assessed 3 times, and the influence of factors influencing the occurrence of delirium was assessed 3 times during the shift. The ISI scale was tested on the sixth day of stay as it examines patients’ insomnia disorders. This was possible in patients who were not sedated.

### 2.5. Inclusion and Exclusion Criteria

Inclusion criteria: Veno-venous ECMO (V-V) or Veno-arterial ECMO (V-A) therapy received, age over 18 years, consent to participate in the study given by the patient or by his/her family (the latter if the patient was unconscious or disoriented at admission). Exclusion criteria: no ECMO therapy received, age below 18 years, no consent given by the patient or their family to participate in the study.

### 2.6. Statistical Analysis

All statistical calculations were carried out using the IBM SPSS 23 statistical package (Armonk, NY, USA) and an Excel 2016 spreadsheet. The statistical analysis included the baseline measurement adjusted to the variables, mean, standard deviation, *N* (%). The following rules were adopted: *p* < 0.05—statistically significant relationship (marked *); *p* < 0.01—highly significant relationship (marked **); and *p* < 0.001—extremely significant relationship (marked as ***).

In order to check if the symptoms of delirium improved, an ANOVA (F) test was used. The level of significance was *p* < 0.05. As a result of the analysis, statistically significant differences in the number of patients with delirium at particular time points were detected (F = 13.229, *p* < 0.00002).

## 3. Results

### 3.1. Participants

**Figure 1:**
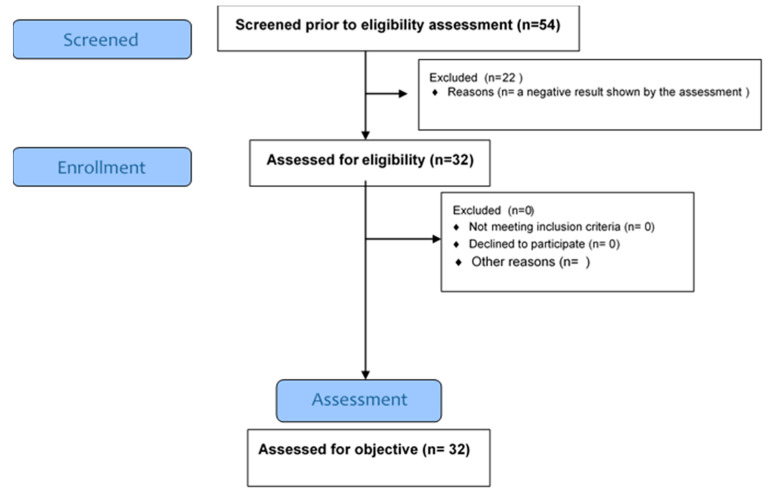
The selection of the group in the study.

The analysis covered all patients enrolled in the ECMO in the period from 2018 to 2020, which means that 54 patients accounted for 100% of those initially enrolled in the study. At the qualification stage based on the NuDesc, 22 individuals were excluded due to a negative result shown by the assessment. Finally, 32 subjects were analyzed—19 women and 13 men (Figure 1). The main reasons for applying the ECMO therapy included circulatory failure (31.25%), cardiopulmonary failure (31.25%), respiratory failure (9.38%), and renal failure (9.38%). The need for respiratory therapy was demonstrated in 25 patients, constituting 78.13% of the sample. During ECMO therapy, direct coercion was applied to 15 patients (47%). In the ECMO group (all patients during ECMO therapy) the distribution was as follows: 44% of the patients had VA and 56% had VV. The distribution of ECMO in both sexes was similar (χ^2^ = 0.09, *p* < 0.75). In the age groups, the ECMO distribution was significantly different (χ^2^ = 4.34, *p* < 0.004). Patients aged 51 and above significantly more often had VV ECMO.

### 3.2. Delirium Syndrome According to NuDESC and DOSS

As a result of the analysis, no statistically significant differences were found in the incidence of delirium in relation to sex, reasons for hospitalization, use of artificial respiration, and airway protection. However, statistical significance was identified in the distribution of these symptoms relative to the application of direct coercion (Table 1). More than half of the patients (59.38%) had delirium symptoms at 12 a.m. and 68.75% at 8 p.m. Delirium was more common during the night.

A post-hoc test (NIR test) examined statistical significance of the differences between assessments at the specific time points. Statistical differences were detected between the first measurement (7 a.m.) and the third measurement (9 p.m.) and between the second (3 p.m.) and the third measurement. The number of patients with probable delirium at 9 p.m. was significantly lower than the number of patients with delirium symptoms at 7 a.m. (*p* < 0.007). The number of patients with probable delirium at 9 p.m. was significantly lower than the number of patients with delirium symptoms at 3 p.m. (*p* < 0.003) (Table 2).

While superimposing the two scales (DOSS and NuDESC), the DOSS assessment at 3 p.m. was disregarded. As a result, the two scales were assessed by superimposing measurements performed at 7 a.m. (DOSS) and 12 a.m. (NuDESC) as well as 9 p.m. (DOSS) and 8 p.m. (NuDESC) (Table 3). Potential incidence of delirium in the studied group was quantitatively measured using DOSS. The assessment at the specific time points determined that symptoms of delirium were present in 27 patients at 7 a.m., 23 patients at 3 p.m., and 9 patients at 9 p.m. (Table 3). The different time of using the scales is related to the guidelines from the authors of the scales.

### 3.3. Delirium Syndrome According to Numeric Rating Scale (NRS)

The NRS describes pain from 0 (no pain), through to 5 (moderate), to 10 (strongest pain imaginable). The patients were rated on the NRS while they were awake and coherent. The average level of pain was 7.16 ± 2, with a median of 8. One in two subjects presented with pain in the range from 5.50 to 9 points. Student’s *t*-test analysis (t) showed a significant effect of sex on the pain severity rating (*t* = 2.78, *p* < 0.01). Women reported significantly (*t* = 2.78, *p* < 0.01) more severe pain (7.89 ± 1.63) than men (6.08 ± 2.06). Respiratory therapy (*t* = 0.65, *p* < 0.52) and airway protection (*t* = –0.15, *p* < 0.88) did not have a statistically significant effect on differences in NRS pain levels.

### 3.4. Delirium Syndrome According to the Richmond Agitation Sedation Scale (RASS) and the Ramsay Sedation Scale (RSS)

The analysis of the distribution of patients in relation to the variables, with the use of chi-squared test of independence, showed there were no statistically significant differences related to sex (χ^2^ = 4.52, *p* < 0.47), use of artificial respiration (χ^2^ = 6.14, *p* < 0.0, 29), and airway protection (χ^2^ = 7.49, *p* < 0.18) relative to the RASS patient condition. The table below presents the characteristics of patient conditions according to the RASS in the case of direct coercion (Table 4).

A continuous infusion of propofol was used in all the patients during the ECMO procedure. The RASS assessment showed that agitated and very agitated patients constituted a majority (37.50% and 18.75%, respectively).

This scale is used in anesthesiology to assess the depth of sedation in ICU patients. Delirium is a state of confusion in the patient. Sedation is a form of drug therapy that can reduce delirium. According to the Ramsay scale, 78.13% of the patients were restless or agitated, while 12.5% of patients were cooperative, tranquil, and oriented. Three patients (9.38%) were sleepy and responded to commands only. The analysis of the distribution of the patients’ condition in the groups of independent variables performed using the chi-squared test showed no statistically significant differences with regard to sex (χ^2^ = 0.61, *p* < 0.73), respiration conducted (χ^2^ = 3.05, *p* < 0.21), and respiratory tract protection (χ2 = 1.84, *p* < 0.39), as well as direct coercion (χ^2^ = 4.07, *p* < 0.13) relative to patient sedation according to Ramsay scale. The chi-squared analysis showed that the distribution of the patients’ condition in the groups specified relative to the sedation rating was significantly different (χ^2^ = 21.21, *p* < 0.02) (Table 5).

### 3.5. Delirium Syndrome According to the Insomnia Severity Index (ISI)

As a result of the chi-squared test analysis, it was found there was a statistically significant effect of direct coercion on the patient’s condition (χ^2^ = 30.74, *p* < 0.001). In the group of patients subjected to direct coercion, some were upset or highly agitated. The average ISI level after 6 days amounted to 13.47 ± 6.14, with a median of 13. The average level in the group of women and men was similar (F = 0.11, *p* < 0.73). The average ISI level after 6 days in the group of younger and older patients was similar (F = 0.13, *p* < 0.71). The distribution of ISI types is presented in the table. The ISI distribution in the group of men and women was similar (χ^2^ = 3.72, *p* < 0.29) and in the age groups the distribution was similar (χ^2^ = 0.31, *p* < 0.95). Table 6 shows the percentage of sleep disorders in patients (Table 6).

### 3.6. Delirium Syndrome According to Thirst Scale

**Figure 2:**
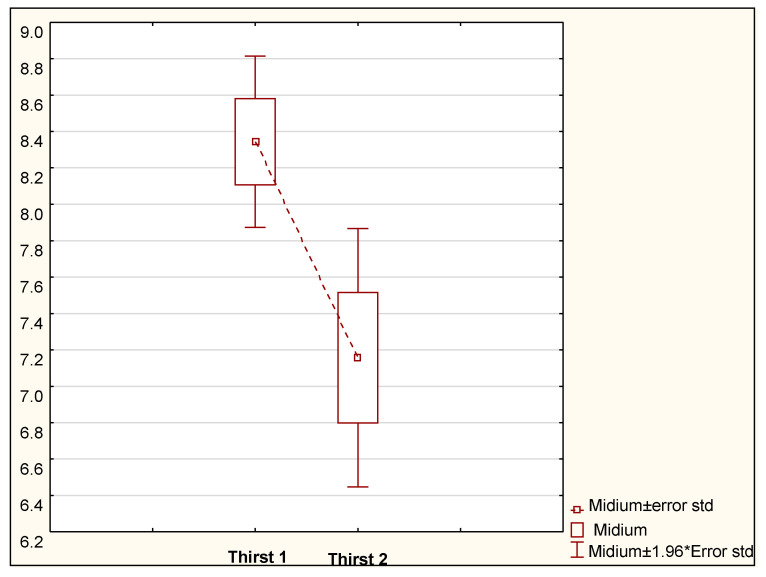
Comparison of average thirst levels before and after administration of an ice cube.

In the study group, thirst intensity was measured on a scale from 0 (no thirst) to 10 (unbearable thirst distress). Before ice cube administration, the thirst intensity rating was 8.34 ± 1.36, with a median of 8. After the ice cube was administered, thirst intensity was significantly lower (*p* < 0.008) at 7.16 ± 2.05 (Figure 2).

The patients who felt thirsty received an ice cube. After eating the ice cube, the patients were again asked to rate thirst intensity.

At a later stage, the mean pain rating and thirst intensity before and after as well as ISI was compared in the delirium groups using Student’s *t*-test. The analyses showed no statistically significant differences in pain ratings and ISI or thirst intensity in the delirium groups (Table 7).

Statistically significant differences were observed in thirst intensity after delirium. In the group of patients with delirium, thirst distress was significantly higher than in the patients who did not have these 2 symptoms at the same time. Later, delirium incidence and delirium distribution were checked relative to RASS, Ramsay scale, the serving of an ice cube, and ISI groups using the chi-squared test. As a result of the analysis, no statistically significant differences were found in the distribution of delirium and delirium incidence relative to groups of these factors (Table 8).

## 4. Discussion

ECMO therapy is often the only solution available for patients with respiratory and circulatory failure, potentially inducing delirium. Research in various aspects of ECMO is limited, and the latest literature does not provide answers to all the problems associated with this therapy [23,24,25]. The main reasons for applying the ECMO therapy in our study included circulatory failure (31.25%), cardiopulmonary failure (31.25%), respiratory failure (9.38%), and renal failure (9.38%). This study aimed to show the incidence of delirium in patients after extracorporeal membrane oxygenation therapy and factors affecting the occurrence of delirium in this group of patients. Due to the severity of ECMO therapy, patients require support at every stage of recovery [1,24]. One of the procedures that may contribute to patient safety in intensive care units involves early identification of delirium, with the use of short scales such as the DOSS and NuDESC. There are many components to patient safety. One of them is peace, which can be of great importance in preventing delirium [1,13]. In our study, more than half of the patients (59.38%) had delirium symptoms at 12 a.m. and 68.75% at 8 p.m. Delirium was more common during the night. Gavinski et al. in their study demonstrated the effectiveness, efficiency, and ease of use of the DOSS as a delirium screening tool [13]. Weckmann and Martin wrote about using DOSS in the ICU [18].

The Delirium Observation Screening Scale is a brief screening tool based on observation. It has been validated in several patient populations, but no published studies took place in the United States or focused on an older, general medicine, inpatient population. Given the low numbers of patients in earlier validation studies, the effectiveness of the DOSS for screening hospitalized, older patients is not yet fully established. DOSS scale is not available in all ICUs in Poland. Statistical differences were detected in our study between the first measurement (7 a.m.) and the third measurement (9 p.m.) and between the second (3 p.m.) and the third measurement. The diagnosis of delirium is often missed, potentiating negative outcomes.

If delirium is detected, the reasons for its occurrence must be known to ensure patient safety. One of the factors investigated in connection to this problem is pain. As reported by other authors, it is difficult to measure pain in unconscious patients and it is even more difficult to predict patients’ reactions once they are awake. Mędrzycka-Dąbrowska et al. describe relationship of pain and various side effects, including delirium [26]. Respiratory therapy (*t* = 0.65, *p* < 0.52) and airway protection (*t* = −0.15, *p* < 0.88) did not have a statistically significant effect on differences in NRS pain levels in our study. This scale is used to examine the patient’s arousal in own study.

Awan et al. in their delirium-related study also used the RASS to check the state of sedation in patients [27]. A continuous infusion of propofol was used in all the patients during the ECMO procedure. The RASS assessment showed that agitated and very agitated patients constituted a majority (37.50% and 18.75%, respectively). In the current study, the patients were first examined for agitation and sedation to move on to other factors. Lewandowska et al. emphasized the role of sleep in the hospital environment in their study [23]. The authors pointed out that sleep disorders are closely linked to the onset of agitation. The present study also discusses the problem of sleep disorders and the effect of agitation on patients undergoing extracorporeal therapy. In their study, Dres et al. focused on sleep disorders during mechanical ventilation, linking two problems, namely extracorporeal circulation and ventilation disorders [28]. In our study, the ISI distribution in the group of men and women was similar (χ^2^ = 3.72, *p* < 0.29) and in the age groups the distribution was similar (χ^2^ = 0.31, *p* < 0.95). Another important element during ECMO therapy is thirst, which often manifests in chronically ventilated patients. In their study discussing a patient’s experience at an intensive care unit, Sung et al. reported that the main complaints of the patient included thirst distress [29]. In line with this, in the present study it has been shown that thirst is likely to contribute to agitation occurring during mechanical ventilation. Our study shows that before ice cube administration, the thirst intensity rating was 8.34 ± 1.36, with a median of 8. After the ice cube was administered, thirst intensity was significantly lower (*p* < 0.008) at 7.16 ± 2.05. At a later stage, the mean pain rating, thirst intensity before and after, and ISI were compared in the delirium groups using Student’s *t*-test. The level of significance was defined at *p* < 0.05. The analyses in this study showed no statistically significant differences in pain ratings and ISI or thirst intensity in the delirium groups. A publication by Krupa et al. showed how important the problem of delirium is during ECMO respiratory and circulatory therapy [30]. There are few reports on how to deal with this problem, but our study suggests that the occurrence of delirium may be prevented by eliminating risk factors. In Poland, in recent years many researchers have made attempts to translate, adapt, culturally validate, and introduce changes and implementations into practice with regard to the Critical Care Pain Observation Tool (CPOT) and Behavioral Pain Scale (BPS) scales [10,11,31]. Further research is needed to strive for the highest quality of patient care in the ICU. Ozga et al. describes the importance of interventions that significantly reduce the risk of delirium in critically ill patients. The coronavirus pandemic carries a high risk of delirium development in various patient groups [32]. Factors influencing the occurrence of delirium in the period from admission to hospital until complete recovery should be sought [33,34]. The study by Ozga et al. showed that nurses face a multitude of limitations due to procedures, lack of equipment, and personnel issues [35].

Study Limitations: There are not many ECMO centers in Poland, which makes it impossible to conduct a larger study on this topic on delirium. The DOSS scale in the literature was used only once in the ICU. There is no training for staff on the use of delirium assessment tools. Delirium is still a huge problem. There is no quality standard for delirium assessment in Poland.

## 5. Conclusions

Delirium in patients undergoing ECMO therapy was confirmed by both the NuDESC and DOSS. Factors such as thirst and sleep disturbance after ECMO therapy have been shown to influence the occurrence of delirium.

Diagnosis of delirium using standardized scales is possible provided that more tests are carried out. Research should be conducted to determine whether the NuDESC scale is equivalent to the DOSS scale.

## 6. Implications for Practice

Delirium is linked to several adverse effects, including pain, thirst, and sleep disorders (by the chi-squared test). To detect delirium, standardized tools such as the DOSS and NuDESC are used. Both scales should be known to nurses working with ECMO patients. It should be emphasized that the current study is the first to take into account patients after ECMO therapy. The study shows that delirium occurs during various therapies used in ICU. Important related issues include the need to promptly diagnose delirium and implement treatment appropriate to the patient’s condition. The use of delirium assessment scales in ICUs should be implemented regardless of the patient’s condition in the ward. Other problems, such as thirst or insomnia, should be assessed regularly in the ICU in addition to delirium.

Further education of nurses and other medical groups in detecting and dealing with delirium is necessary. The competency of nurse teachers and collaboration between nurse educators, nurse leaders, and mentors are crucial for achieving high quality evidence-based nursing education [32,35].

## Figures and Tables

**Table 1:** Characteristics of the distribution of delirium syndrome according to the Nursing Delirium Screening Scale (NuDESC).

NuDESC	12 a.m.		8 p.m.		*p*
	N	%	N	%	
<2 no delirium	13	40.63%	10	31.25%	0.40933
≥2 delirium	19	59.38%	22	68.75%	0.40412
All	32	100%	32	100%	

N = responders; *p*-value < 0.05.

**Table 2:** Characteristics of groups according to the Delirium Observation Screening Scale (DOSS) in the examined group.

DOSS	7 a.m.		3 p.m.		9 p.m.		*p*
	N	%	N	%	N	%	
<3 no delirium	5	15.63%	9	28.13%	23	71.88%	0.000000
≥3 possible delirium	27	84.38%	23	71.88%	9	28.13%	0.000016
All	32	100%	32	100%	32	100%	

N = responders; *p*-value < 0.05.

**Table 3:** Characteristics of delirium occurrence in groups of direct coercion.

The Use of Direct Coercion							
DOSS/Nu Desc	No		Yes		8 p.m.–9 p.m.		*p*
	*N*	%	*N*	%	*N*	%	
No delirium	15	100.00%	11	64.71%	26	81.25%	0.40933
Delirium	0	0.00%	6	35.29%	6	18.75%	0.40412
All	15	100.00%	17	100.00%	32	100%	

*N* = responders; *p*-value < 0.05.

**Table 4:** Characteristics of patient states on the Richmond Agitation Sedation Scale (RASS) during direct coercion.

	7 a.m.				3 p.m.				9 p.m.			
Patient’s Condition in RASS	Yes		No		Yes		No		Yes		No	
	*N*	%	*N*	%	*N*	%	*N*	%	*N*	%	*N*	%
Combative +4	0	0.00%	0	0.00%	0	0.00%	0	0.00%	0	0.00%	0	0.00%
Very agitated +3	0	0.00%	6	18.75%	2	6.25%	4	12.50%	5	15.63%	1	3.13%
Agitated +2	0	0.00%	12	37.50%	1	3.13%	11	34.38%	9	28.13%	3	9.38%
Restless +1	1	3.13%	6	18.75%	5	15.63%	2	6.25%	6	18.75%	1	3.13%
Alert and calm 0	0	0.00%	0	0.00%	0	0.00%	0	0.00%	0	0.00%	0	0.00%
Drowsy –1	1	3.13%	1	3.13%	1	3.13%	1	3.13%	1	3.13%	1	3.13%
Light sedation –2	1	3.13%	1	3.13%	0	0.00%	2	6.25%	1	3.13%	1	3.13%
Moderate sedation –3	2	6.25%	1	3.13%	0	0.00%	3	9.38%	1	3.13%	2	6.25%
Deep sedation –4	0	0.00%	0	0.00%	0	0.00%	0	0.00%	0	0.00%	0	0.00%
Unarousable sedation –5	0	0.00%	0	0.00%	0	0.00%	0	0.00%	0	0.00%	0	0.00%
**Altogether**	**12.63164**	**df = 5**	***p* = 0.02709**		**12.35372**	**df = 5**	***p* = 0.03025**		**4.015653**	**df = 5**	***p* = 0.54716**	

*N* = responders; df—distribution free; *p*-value < 0.05.

**Table 5:** RASS patient status in the Ramsey Sedation Grade groups.

Patient Condition by the RASS	Degree of Sedation by Ramsey						All	
	Anxious and Agitated Patient		Cooperative, Tranquil, Oriented Patient		Sleeping Patient Responsive only to Instructions			
	*N*	%	*N*	%	*N*	%	*N*	%
Very upset	6	24%	0	0%	0	0%	6	18.75%
Upset	10	40%	2	50%	0	0%	12	37.50%
Restless	5	20%	2	50%	0	0%	7	21.88%
Sleepy	0	0%	0	0%	2	66.67%	2	6.25%
Light sedation	1	4%	0	0%	1	33.33%	2	6.25%
Moderate sedation	3	12%	0	0%	0	0%	3	9.38%
Altogether	25	100%	4	100%	3	100%	32	100.00%

*N* = responders; RASS = Richmond Agitation and Sedation Scale.

**Table 6:** Characteristics of sleep disorders after 6 days.

ISI Scale	*N*	%
0–7 = No clinically significant insomnia	8	25%
8–14 = Subclinical insomnia	9	28%
15–21 = Clinical insomnia (moderate severity)	10	31%
22–28 = Clinical insomnia (severe)	5	16%
All	32	100%

*N* = responders; ISI = insomnia severity index.

**Table 7:** Characteristics of the *t*-Student test indicator and their significance (*p*) for comparison of delirium and delirium groups.

Occurrence of Delirium	*t*	*p*
Pain (NRS)	−0.68	0.50
Thirst before ice cube	0.68	0.50
Thirst after ice cube	−2.11	0.04
ISI	2.01	0.05

NRS = numeric rating scale; ISI = insomnia severity index; *t* = *t*-Student test; *p* value < 0.05.

**Table 8:** Chi-squared test indicators and their statistical significance (*p*).

Occurrence of Delirium	chi	*p*
RASS	8.89	0.11
Ramsey	4.22	0.12
Ice cube	0.83	0.36
ISI (Insomnia Severity Index)	4.87	0.18

RASS = Richmond Agitation and Sedation Scale; ISI = insomnia severity index; chi = Chi-squared test; *p* value < 0.05.

## Data Availability

Not applicable.

## Supplementary Materials

The following are available online at https://www.mdpi.com/article/10.3390/ijerph18084029/s1, File S1: STROBE Statement—checklist of items that should be included in reports of observational studies.
